# Intra-arterial lidocaine administration during uterine fibroid embolization to reduce the immediate postoperative pain: a prospective randomized study

**DOI:** 10.1186/s42155-020-0099-4

**Published:** 2020-02-10

**Authors:** Stevo Duvnjak, Poul Erik Andersen

**Affiliations:** 1 Department of Radiology, Odense University Hospital, Sdr. Boulevard 29, 5000 Odense, DK Denmark; 2 Institute for Clinical Research, University of Southern Denmark, Odense, Denmark

**Keywords:** Uterine fibroid embolisation, Intraarterial lidocaine, Pain after embolisation, Post-embolization syndrome

## Abstract

**Background:**

To investigate if intra-arterial lidocaine administrated immediately after the embolisation endpoint reduces the pain.

**Methods:**

Forty patients were randomised and 36 completed the study for purposes of analysis. In one group, the patients got 1% 10 ml lidocaine (100 mg) administered into each uterine artery immediately after embolisation with microspheres. The other group was embolised without supplementary lidocaine. The patients scored their pain on a visual analogue scale (VAS) 2 h, 4 h, 7 h, 10 h and 24 h after embolisation, and the total amount of used morphine was noted. Three-month follow-up MRI control was scheduled for all the patients to investigate the infarction rate.

**Results:**

Embolisation was performed without any complications and with embolisation of both uterine arteries in all cases. Intra-arterial lidocaine was administered in all 20 patients without complications, and 20 patients in a control group did not receive lidocaine intra-arterial. VAS schemes showed a significant reduction in pain experience 2 h after UFE where mean pain score in the lidocaine group was 42.7 ± 21.4 compared with the control group in which the mean pain score was 61.1 ± 20.4 (*p* < 0.02). There was no significant difference in pain score 4 h, 7 h, 10 h and 24 h after UFE. In the lidocaine group, the mean amount of used morphine was significantly less with 11.2 mg compared with 20.2 mg in the control group (*p* < 0.03). Three months of MR follow-up control showed no significant difference in the grade of fibroid infarction.

**Conclusion:**

Intra-arterial Lidocaine administration after embolisation is safe and effective in reducing post-procedural pain in the early hours and opioid usage in the first 24 h following UAE.

## Introduction

Uterine fibroid embolisation (UFE) is a well-described and well-established interventional radiology procedure to treat symptomatic uterine fibroids. The efficacy and long term outcome is documented in several randomised studies and long-term follow-up results are confirmed (Hehenkamp et al. 2008; Scheurig-Muenkler et al. 2013; The REST Investigators. 2007). Pain immediately after UFE is a well known adverse effect of the treatment and is the most frequent patient complaint (Spencer et al. 2013). The pain is believed to occur due to temporary myometrium ischemia after embolisation. The pain usually starts 1 hour after UFE and increases during the following 5–7 h (Spencer et al. 2013). After that period, the pain usually decreases and the majority of patients are discharged within 24 h after UFE without pain. Few studies are describing that intra-arterial lidocaine can reduce the pain during embolisations including UFE (Noel-Lamy et al. 2017; Keyoung et al. 2001; Lee et al. 2001). Another more invasive analgesic technique to reduce or eliminate embolization-induced ischemic pain is hypogastric nerve blockade which has shown promising results (Binkert et al. 2015; Yoon et al. 2018).

The present study aims to investigate if intra-arterial lidocaine administrated in the uterine arteries immediately after UFE reduces the pain.

## Materials and methods

Institutional board and ethical committee approval were obtained (S-345678). Between August 2017 and July 2019, all patients with symptomatic uterine fibroids who accepted participation were block randomised were smaller blocks of fewer patients randomised to the two treatments at a time intending to have 20 patients in each group. The patients in one group had 1% 10 ml lidocaine (100 mg) administrated intra-arterially immediately after embolisation in each uterine artery, so the total doses given to each patient was 200 mg of lidocaine. In the other (control) group of patients, the procedure was performed according to standard principles without supplementary injection of lidocaine. The patient’s age and symptoms, fibroid number, localisation and total uterine and dominant fibroid volume changes before and after an embolization were analysed.

The standard embolisation technique was performed in local anaesthesia via either transfemoral or transradial access using diagnostic 5F catheter advanced into the internal iliac artery. Further, in all cases, micro-catheter (Direxion hi-flow 0.027- in., Boston scientific Massachusetts MA, USA) was used and advanced into the horizontal part of the uterine arteries. Embolisation with microspheres tris-acryl gelatin (500–900 μm) (Embosphere, Biosphere Medical, Paris, France) to near stasis defined as slow forward flow trough the main uterine artery with at least five heartbeats for clearance of contrast from uterine artery with the pruning of peripheral vessels. The total amount of the used microspheres was recorded and compared between the groups. All patients had patient-controlled analgesia pump (PCA) loaded with 2 mg morphine sulphate and an intermittent dose of 1 mg morphine with lockout intervals of 10 min. Before the beginning of the embolisation, a loading dose of 2 mg morphine was given to all patients. The patients were instructed to push the button of the PCA for the first time before the pain developed. No antibiotics were given and no urinary catheter was deployed. There was no standardised regimen concerning usage of adjunctive analgesia and sedation, and if needed, some patients take additional drugs such as non-steroid anti-inflammatory medications on the ward individually.

Inclusion criteria were symptomatic fibroids in a premenopausal woman with bleeding and/or bulky symptoms. Size and number of fibroids were not exclusion criteria. Patients who did not want to participate in the study were excluded. Allergy to morphine or lidocaine, heart block and active pelvic infection were exclusion criteria. After embolisation, the patients were transferred to the ward and a visual analogue pain scale (VAS) with oral and written instructions was given to the patients (Bijur et al. 2001). The patients marked their sensation of pain on the VAS at 2 h, 4 h, 7 h, 10 h and 24 h after embolisation. VAS schemes were collected the following day before discharge and the total amount of used morphine was registered as well. Three-month follow-up MRI control follow-up was scheduled for all the patients to investigate the infarction rate percentage using the description defined as fibroma infarction of 100%, 90–99%, or below 90% (Duvnjak et al. 2017). Infraction, less than 90% of total fibroid burden, was defined as insufficient.

Descriptive statistics were used for baseline patients and fibroid characteristics and were presented as number and percentage and as mean and standard deviation (SD). The student’s t-test was used for comparison between the groups regarding the used amount of morphine and pain experience. *P*-value < 0.05 was considered statistically significant. SPSS software package (Statistics 21, IBM Corporation, Armonk, NY, USA) program was used for analysis.

## Results

**Fig. 1:**
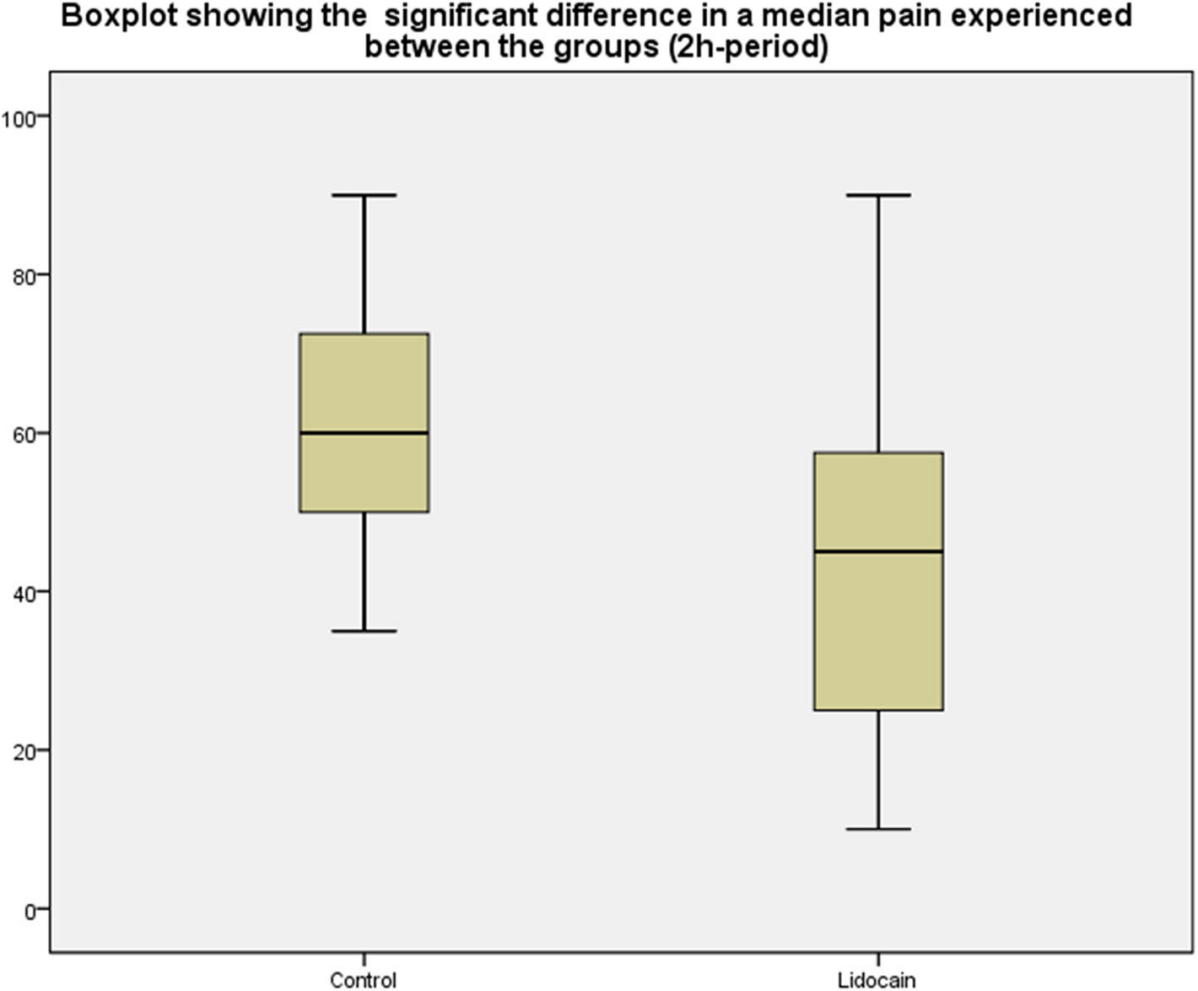
Boxplot showing the significant difference in a median pain

Fifty-four consecutive patients were treated with embolisation in the period August 2017 –July 2019 at the Department of Radiology. Fourteen patients did not want to participate in the study and were excluded. There were no patients with allergy to lidocaine or morphine or with heart problems. Thus, in all 40 patients were included in the study and all gave written consent. In all patients, UFE was performed without complications and with embolisation of both uterine arteries as intended. The demography of the patients, baseline fibroid description and patient symptoms are presented in Table 1. There was no statistically significant difference between the lidocaine treated group and the control group in terms of age (*p* < 0.78), fibroid numbers (*p* < 0.89), fibroid location (*p* < 0.22), dominant symptoms (*p* < 0.54), or amount of used microspheres *p* < (0.19). Intra-arterial lidocaine was administrated in all 20 patients without complications and was well tolerated. VAS schemes showed a significant difference in pain experienced between the groups 2 h after UFE. The median pain experienced in the lidocaine group was 45 ± 21.1 compared with the control group 60.1 ± 15.2 (*p* < 0.02) (Fig. 1). In the control group, four patients were not included in the analysis due to either withdrawal from the study (two patients) or due to not properly filled-in VAS schemes (two patients). There was no difference in pain experience, 4 h, 7 h, 10 h, and 24 h after UFE *p* < 0.06, *p* < 0.83, *p* < 0.61, *p* < 0.15). The total amount of used morphine was recorded 24 h after UFE. In the lidocaine group, the mean amount of used morphine was 11.2 mg compared with 20.2 mg in the control group (*p* < 0.03). The detailed scores of the pain experience and comparison between the groups and the use of morphine are presented in Table 2. All patients were discharged about 24 h after UFE and continued on non-steroid anti-inflammatory medication. Three patients (two in the lidocaine group and one in the control group) phoned back due to pain experienced on day three after UFE, but there were no re-admissions to hospital after discharge. No complications or readmissions occurred during 3 months of follow-up in any group. Three-month follow-up MRI control was obtained in 36 patients and showed no significant difference in terms of fibroid infarction between the groups (Table 3).

**Table 1:** Pre-embolization patients and fibroid characteristics

	Lidocaine group	Control group	*p*-value
Mean age (years-SD)	44.4 ± 3.3	43.1 ± 5.11	*p* < 0.78
Dominant fibroid volume (mean-SD)	183.1 ± 206.5 cm^3^	262.8 ± 188.7 cm^3^	*p* < 0.26
Singular fibroid	*n* = 9 (45%)	*n* = 7 (44%)	
2–5 fibroids	*n* = 4 (20%)	*n* = 6 (37%)	
> 5 fibroids	*n* = 7 (35%)	*n* = 3 (19%)	
Dominant symptom			*p* < 0.54
Bleeding symptoms	*n* = 11 (55%)	*n* = 6 (37%)	
Bulk symptoms	*n* = 5 (25%)	*n* = 7 (44%)	
Both bleedings and bulk symptoms	*n* = 4 (20%)	*n* = 3 (19%)

**Table 2:** The pain experience and used of morphine -comparison between the groups

	Lidocaine group	Control group	*p*-value
Pain experience 2 h after UFE (mean-SD)	42.7 ± 21.4	61.1 ± 20.4	***p*** **< 0.02**
Pain experience 4 h after UFE (mean-SD)	39.7 ± 23.1	53.7 ± 21	*p* < 0.06
Pain experience 7 h after UFE (mean-SD)	41.5 ± 20.8	42.8 ± 15.4	*p* < 0.83
Pain experience 10 h after UFE (mean-SD)	34.7 ± 19.6	31.8 ± 12.5	*p* < 0.61
Pain experience 24 h after UFE (mean-SD)	20.7 ± 14.5	27.3 ± 12.5	*p* < 0.15
Total amount of used morphine mean value (mg-SD)	11.2 ± 7.3 (range: 4–32)	20.2 ± 10.8 (range:10–44)	***p*** **< 0.03**

**Table 3:** Detail about used microspheres and MR three months control results

	Lidocaine group	Control group	*p*-value
The total amount of used microspheres (ml- SD)	6.9 ± 2.5	9.7 ± 2.8	*p* < 0.19
Dominant fibroid volume reduction (three months control)	84.4 ± 92 cm^3^	145.1 ± 175 cm^3^	*p* < 0.15
Total uterine volume	161,7 ± 154 cm^3^	261,9 ± 288 cm^3^	*P* < 0.26
Total fibroid burden infarction			*p* < 0.86
100% or 90–99%-fibroid infraction	18 (94%)	14 (95%)	
< 90% fibroid infarction	2 (6%)	2 (5%)	

## Discussion

The present randomised study confirmed that intra-arterial lidocaine administration after UFE reduce the pain in the first hours after embolisation. Lidocaine has a half-life about 90–120 min, and the effect was confirmed in this study during the first 2 hours after UFE. Noel-Lamy et al. 2017, showed in prospective study similar results with the maximal effect and pain control in within two to 4 hours after UFE. In another study, pain control was achieved in a longer period after UFE but the methodology was not the same as in the present study (Kim et al. 2008). Katsumori et al. 2020, in his study, did not achieve significant statistical difference comparing the patients who received intra-arterial lidocaine and those who did not get the lidocaine. This study was retrospective including the patients treated with embolisation from 2014 to 2019. The amount of used lidocaine was 80 mg comparing to our study, where 200 mg lidocaine was used that can probably explain the different outcome. Zhan et al. 2005, used in his study 40 mg od lidocaine and showed significant pain reduction despite the small amount of the drug in the patients who received lidocaine but the used microparticles and time to record pain was different than in our study.

Further, Katsumori et al. 2020 recorded the VAS pain during the embolisation and first, 3 hours after the intervention. The maximal effect of intra-arterial lidocaine is in the first 2 hours due to the half-life of the drug.

The pain experienced in our study was lower compared with the control group after 4 h,7 h,10 h, and 24 h but without statistical significance, similar to Katsumori et al. 2020 study confirming that lidocaine has the maximal effect in the first 2 hours.

The intra-arterial lidocaine administration was safe in all patients without any complaints and adverse events and was easy and safe to deliver after the embolisation endpoint was achieved. In previously published studies, there were no reported cases of adverse reactions during the intraarterial lidocaine administration as well (Noel-Lamy et al. 2017; Keyoung et al. 2001; Lee et al. 2001). When the embolisation endpoint is achieved, injection of lidocaine might theoretically reflux backwards into other vessels, but it seems not to be the case as present investigation demonstrates that this technique works, but this is a subjective opinion. In this study embolisation endpoint was sluggish forward flow and we administrated lidocaine over 30–60 s very slowly. Control MR imaging showed similar results compared with a control group allowing us to conclude that this technique is efficient. The administration of lidocaine before or together with microspheres might reduce the fibroid infarction rate and thus not end-up with an optimal result and therefore is not recommended (Noel-Lamy et al. 2017; Keyoung et al. 2001). All patients had a PCA pump allowing us to record the total amount of morphine which is an advantage compared with another similar study (Noel-Lamy et al. 2017). Hypogastric nerve blockade is invasive and according to the literature not complicated to learn (Binkert et al. 2015). However, intraarterial lidocaine administration is a simpler less-invasive technique than hypogastric nerve blockade with a likely lesser learning curve and could be widely adopted more easily. Therefore, very simple intraarterial lidocaine administration in total doses of 200 mg is preferable in our opinion despite the limited long-term pain effect due to lidocaine metabolism. A recent published systemic review emphasises the need for more evidence, and no clear advantage of any of the used methods for pain control after UFE have been demonstrated so far (Saibudeen et al. 2019).

The limitation of this study are small number of the patients, not double-blinded design and drop-out of four patients that withdrew or did not fill in the VAS schema properly. Further, different access via transfemoral or transradial access could be a possible confounding factor due to early mobilisation after transradial access and eventualy use of additional non-steroid anti-inflammatory drugs in the immediately post-embolisation period. Finally, the specific size and volume of used embosphere was not standardised or analysed systematically.

## Conclusion

Intra-arterial Lidocaine administration after embolisation is safe and effective in reducing post-procedural pain in the early hours and opioid usage in the first 24 h following UAE. Further studies of how further to reduce pain are warranted.

## Data Availability

The datasets analyzed during the current study are available from the corresponding author on reasonable request.

## Abbreviations

F: French
MR: Magnentic resonance
PCA: Patient-controlled analgesia pump
UFE: Uterine fibroid embolization
VAS: Visual analogue scale
